# Analysis of Keratoconus-Related Phenotypes in Two Pcsk1 Mouse Models

**DOI:** 10.1167/tvst.15.2.3

**Published:** 2026-02-04

**Authors:** Carol Beatty, Jingwen Cai, Hongfang Yu, Jiong Sun, Yejin Heo, Keith H. Baratz, Ashlie A. Bernhisel, Sanjay V. Patel, Amy J. Estes, Anthony N. Kuo, Yutao Liu

**Affiliations:** 1 Medical College of Georgia, Augusta University, Augusta, GA, USA; 2 Department of Cellular Biology and Anatomy, Augusta University, Augusta, GA, USA; 3 Department of Ophthalmology, Mayo Clinic, Rochester, MN, USA; 4 Department of Ophthalmology, Augusta University, Augusta, GA, USA; 5 James and Jean Culver Vision Discovery Institute, Augusta University, Augusta, GA, USA; 6 Department of Ophthalmology, Duke University Medical Center, Durham, NC, USA; 7 Center for Biotechnology and Genomic Medicine, Augusta University, Augusta, GA, USA

**Keywords:** keratoconus (KC), PCSK1, genetics, cornea

## Abstract

**Purpose:**

Previously, a variant within the *Pcsk1* gene was found to segregate with the keratoconus (KC) phenotype in whole genome sequencing of a four-generation family. We aimed to evaluate a potential relation between the *Pcsk1* gene and corneal phenotype in mouse models.

**Methods:**

Two strains of *Pcsk1* mice, one with a knockout (KO) and one with an N222D point mutation, were bred. Central corneal thickness (CCT) was determined using spectral domain optical coherence tomography (SD-OCT) in PC1/3^+/+^ (*n* = 12), PC1/3^+/K^^ (*n* = 14), PC1/3^K^/K^^ (*n* = 5), PC1^+/+^ (*n* = 8), PC1^+/ N222D^ (*n* = 15), and PC1 ^N222D / N222D^ (*n* = 7) mice at 3 and 6 months of age. Pachymetry maps were generated using the Mouse Corneal Analysis Program (MCAP) to process OCT images. Hematoxylin and eosin (H&E) staining using corneal sections from these animals were used to examine morphological changes.

**Results:**

No significant differences in corneal CCT, pachymetry, or morphology were observed among any of the mutant mice compared with their control littermates.

**Conclusions:**

In this setting, neither the N222D point mutation nor the Pcsk1 KO affected the corneal phenotype in two mouse models. The *Pcsk1* gene could contribute to keratoconus when paired with additional genetic and environmental factors, not included in this study.

**Translational Relevance:**

Genetic factors are known to contribute to the pathogenesis of keratoconus. Although a variant in the *Pcsk1* gene has been shown to segregate with keratoconus in a family, there is yet no evidence of *Pcsk1* gene mutation correlating with pathogenic corneal phenotype in mouse models.

## Introduction

Keratoconus (KC) is a bilateral, asymmetric corneal ectasia characterized by irregular thinning and steepening of the cornea into a conical shape.^1^^–^^3^ These progressive changes in the cornea dramatically affect vision, leading to irregular astigmatism, myopia, and decreased visual acuity.^4^^–^^6^ Symptoms most often arise in the second or third decade of life, progress into adulthood, and stabilize in the third or fourth decade.^7^^–^^9^ KC is a multifactorial disease influenced by genetic, environmental, hormonal, and inflammatory factors.^10^^–^^14^ Many risk factors and comorbid conditions are strongly linked to KC.^3^ Yet, the mechanisms, etiology, and underlying genetic basis of KC remain unclear.^4^^,^^10^^,^^15^ There is a significant need to characterize the pathogenesis of KC to identify patients at risk, diagnose the condition earlier, develop therapeutic targets, and improve outcomes for affected patients.^16^

Although some cases of KC present sporadically, genetic factors have been correlated with KC risk.^10^^,^^17^ Family-based linkage studies have identified several genomic regions.^10^^,^^17^ KC is associated with several genetic conditions, including Down syndrome,^18^ Leber congenital amaurosis,^19^ and connective tissue disorders.^20^ Having a first-degree relative with KC is consistently identified as a major risk factor.^21^ The prevalence of KC among relatives of patients with the disease (3.34%) is 15 to 67 times higher than in the general population (0.05%–0.23%).^22^

Family-based genome-wide linkage studies have identified several loci for the disease.^10^^,^^21^^,^^23^ Interestingly, linkage loci in the region of chromosome 5q14.1–q21.3 were identified in multiple independent studies.^24^^–^^26^ Further exploration of the 5q14.1–q21.3 region focused on a 4-generation Caucasian family with autosomal-dominant KC. Using whole exome sequencing (WES), Khaled et al. identified potentially pathogenic variants in the *PPIP5K2* gene.^27^ Subsequently, Akoto et al. suggested that the *PPIP5K2* gene may be relevant to KC development in mouse models.^28^ Khaled et al. additionally utilized whole genome sequencing (WGS) in the four-generation family and identified an intronic variant within the *Pcsk1* gene (rs373951075, c.1096-10G>A) with complete segregation of the KC haplotype. It was hypothesized that this intronic variant in *Pcsk1* may work in tandem with *PPIP5K2* to determine clinical features associated with KC.^27^ In this study, we will focus on the *Pcsk1* gene and further explore its potential relationship to KC in mouse models with Pcsk1 knockout (KO; complete loss of function) or an N222D point mutation (partial loss of function).^29^^–^^31^ These two mouse strains carrying a complete KO or a point mutation were selected for their resemblance to partial and complete loss of Pcsk1 protein function in human patients, spanning a wide range of Pcsk1 expression and activities.

## Methods

### Mouse Husbandry

The mouse protocol adhered to the ARVO Statement for the Use of Animals in Ophthalmic and Vision Research and was approved by the Institutional Animal Care and Use Committee (IACUC) at Augusta University. Breeding pairs of heterozygous C57BL/6J-*Pcsk1^tm1Dfs^* (stock #006327) and C57BL/6J-*Pcsk1^N222D^* (stock #006699) mice were obtained from the Jackson Laboratory (Bar Harbor, ME), maintained, and genotyped as previously described.^27^^,^^31^^,^^32^

For the *Pcsk1^tm1Dfs^* mouse model, a neomycin selection cassette with an upstream 3′ splice site and a downstream transcriptional termination sequence with a poly(A) tail was inserted to replace exon 1 and several transcription control elements. The mutation causes the absence of PC1/3 transcript and protein in homozygous *Pcsk1^tm1Dfs^* mutant mice, leading to postnatal growth impairment and defects in processing hormone precursors, such as growth hormone-releasing hormone (GHRH).^32^

The *Pcsk1^N222D^* mouse models were generated by ENU-induced point mutation. The novel mouse PC1 allele (N222D) leads to obesity and abnormal proinsulin processing without insulin resistance or diabetes.^31^ This mutated PC1 (N222D) protein is largely mislocalized and undergoes more efficient degradation via the ubiquitin-proteasome system than the wildtype protein.^33^

These two mutant mouse models mimic distinct effects of *Pcsk1*. The PC1/3 (*Pcsk1^tm1Dfs^*) model mimics gene KO, whereas the PC1 (*Pcsk1^N222D^*) model mimics partial loss. For the remainder of this article, the mutant models will be specified using their common names as shown in the Table. Knowing that KC most commonly develops in humans’ second or third decade of life, the 3- and 6-month endpoints were chosen as they correlate to 15 and 30 years of age in humans,^34^ respectively.

**Table.** Summary of Mouse Strain Information

Common Name	Genotype	PCSK1 Function
PC1/3^+/+^	*Pcsk1^+/+^*	Wildtype
PC1/3^+/K^^	*Pcsk1^+ /tm1Dfs^*	Heterozygous knock out
PC1/3^K^/K^^	*Pcsk1^tm1Dfs/tm1Dfs^*	Homozygous knock out
PC1^+/+^	*Pcsk1^+/+^*	Wildtype
PC1^+/N222D^	*Pcsk1^+/N222D^*	Heterozygous partial loss
PC1^N222D/N222D^	*Pcsk1^N222D/N222D^*	Homozygous partial loss

### Spectral Domain Optical Coherence Tomography

At 3 and 6 months of age, the anterior segment was visualized using spectral domain optical coherence tomography (SD-OCT) with a Bioptigen Envisu-R2200 Spectral Domain Ophthalmic Imaging System (Leica Microsystems, Wetzlar, Germany).^27^^,^^35^ Mice were briefly anesthetized with a cocktail containing 30 mg/mL xylazine (NDC code 59399-110-20) and 100 mg/mL ketamine (NDC code 11695-0703-1; Covetrus, Portland, ME) at a dosage of 10 µL/g of mouse weight. GenTeal Lubricant Eye Gel and Systane eye drops (Alcon, Fort Worth, TX) were used to prevent mouse cornea dryness. A 12-cm telecentric probe was used, with the reference arm set to position 245 for corneal imaging. The central corneal thickness (CCT) was measured for each mouse using a digital caliper tool available in the InVivoVue software V2.2 (Leica Microsystems, Wetzlar, Germany) as described previously.^27^^,^^28^ Briefly, the frame of the central cornea was located with the display line on the display volume intensity projection window, where an orthogonal aiming scan of the mouse eye was demonstrated. The caliper was locked in the vertical direction, and its top edge was placed on the apex of the cornea, while the bottom edge was extended to the bottom-most surface of the cornea to determine the CCT.

### Corneal Thickness Mapping

Corneal thickness mapping was performed using OCT scanning profiles from two strains of *Pcsk1* mice, as previously described.^27^^,^^28^ Briefly, the mouse cornea analysis program (MCAP) software was used to determine OCT-based corneal pachymetry of the mice. Each corneal OCT scan was segmented to determine the corneal endothelial and epithelial surfaces using a semi-automated algorithm extrapolating from a user-drawn seed segmentation of the first frame. The remaining frames were then auto-segmented using the user-drawn seed. The algorithm accounts for axial motion correction, refraction differences, and optical path length distortions. Finally, direct z-axis subtraction of the epithelial and endothelial corneal layers was conducted to create pachymetry maps of each mouse cornea.

### Corneal Histology

Mice were euthanized for histology at 6 months old according to the approved protocol by the IACUC at Augusta University. The eyeballs were collected, fixed for 24 hours in Davidson's fixative, and transferred to 70% ethanol. After the specimens were paraffin-embedded, the eyeballs were cut through the optic nerve head (ONH) and the center of the cornea in a sagittal plane. Three sections per eyeball with a thickness of 5 µm were processed at the AU Electron Microscopy and Histology Core (RRID: SCR_026810) for hematoxylin and eosin (H&E) staining. The corneal images were scanned and analyzed with the Axioscan 7 Digital Slide Scanner (Zeiss, Oberkochen, Germany) in the Cell and Tissue Imaging Core facility (RRID: SCR_026799) at Augusta University.

### Statistical Analyses

CCT differences between mice with different genotypes of PC1/3 or PC1 strains were assessed using 1-way ANOVA with the Kruskal-Wallis (nonparametric) test. Significance was defined as *P* value ≤ 0.05. Analyses were performed using GraphPad (GraphPad Software, San Diego, CA).

## Results

The analyzed cohorts included 61 male and female mice. Of the 31 male/female mice bred from the *Pcsk1^tm1Dfs^* model, there were 12 wildtype mice (*PC1/3^+/+^*, 7 male mice and 5 female mice), 14 heterozygous (*PC1/3^+/K^^*, 9 male mice and 5 female mice), and 5 homozygous (*PC1/3^K^/K^^*, 1 male mouse and 4 female mice). Of the 30 male/female mice bred from the *Pcsk1^N222D^* model, there were 8 wildtype (*PC1^+/+^*, 4 male mice and 4 female mice), 15 heterozygous (*PC1^+/N222D^*, 10 male mice and 5 female mice), and 7 homozygous mice (*PC1^N222D/N222D^*, 4 male mice and 3 female mice).

### CCT in Pcsk1 Animal Models

**Figure 1.**
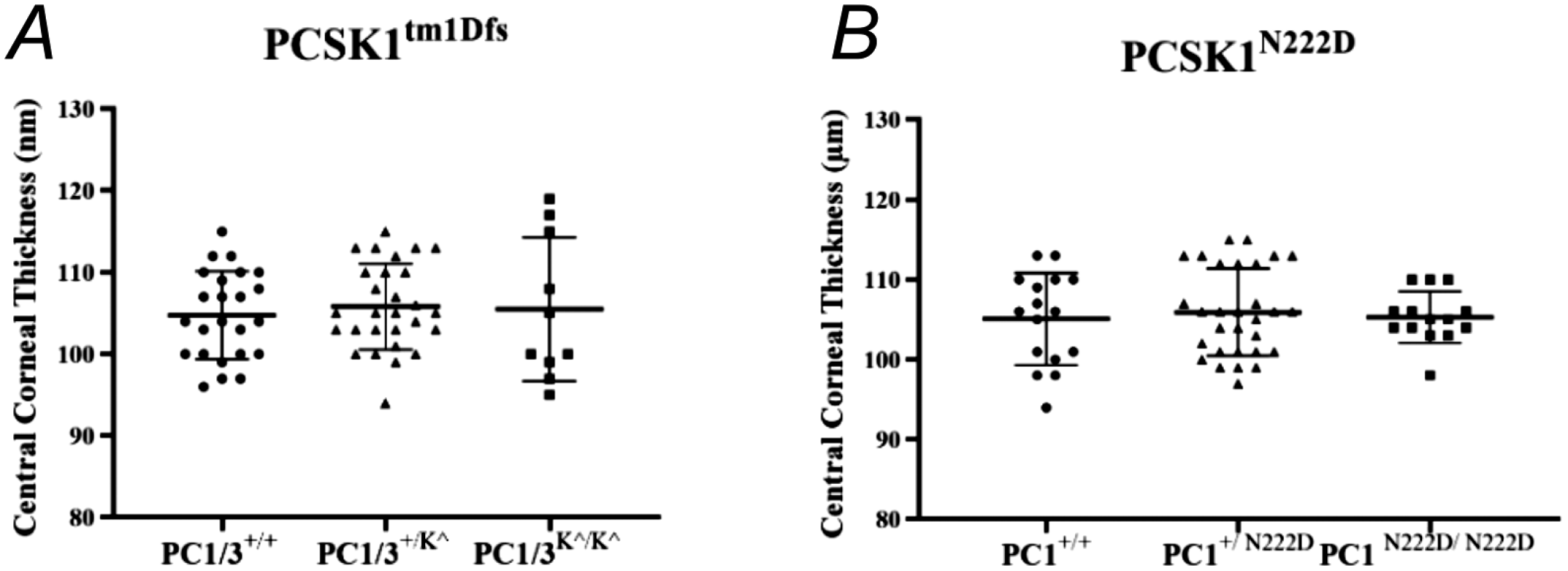
CCT measurements in two Pcsk1 strains. CCT in (**A**) *PC1/3^+/+^* wildtype (*n* = 12), *PC1/3^+/K^^* heterozygous (*n* = 14), and *PC1/3^K^/K^^* homozygous (*n* = 5). No significant CCT differences were observed among the three genotypes (1-way ANOVA with Kruskal-Wallis test, *P* value 0.7329). (**B**) *PC1^+/+^* wildtype (*n* = 8), *PC1^+/ N222D^* heterozygous (*n* = 15), and *PC1 ^N222D/ N222D^* homozygous (*n* = 7). No significant CCT differences were identified among the three genotypes (1-way ANOVA with Kruskal-Wallis test, *P* value = 0.9049).

We sought to identify CCT differences between mice with different genotypes using OCT data. No significant differences in CCT were observed at 6 months in the *PC1/3* KO mice (1-way ANOVA with Kruskal-Wallis test, *P* value = 0.7329) or *PC1* mutant mice (1-way ANOVA with Kruskal-Wallis test, *P* value = 0.9049) when compared with their wildtype littermates (Figs. 1A, 1B). We also measured many mice at 3-month age and observed no significant CCT differences in the KO PC1/3 or mutant PC1 mice when compared with the wildtype mice (Supplementary Fig. S1; 1-way ANOVA with Kruskal-Wallis test, *P* value = 0.9618 and 0.9856, respectively).

### OCT Evaluation in Pcsk1 Mouse Models

**Figure 2.**
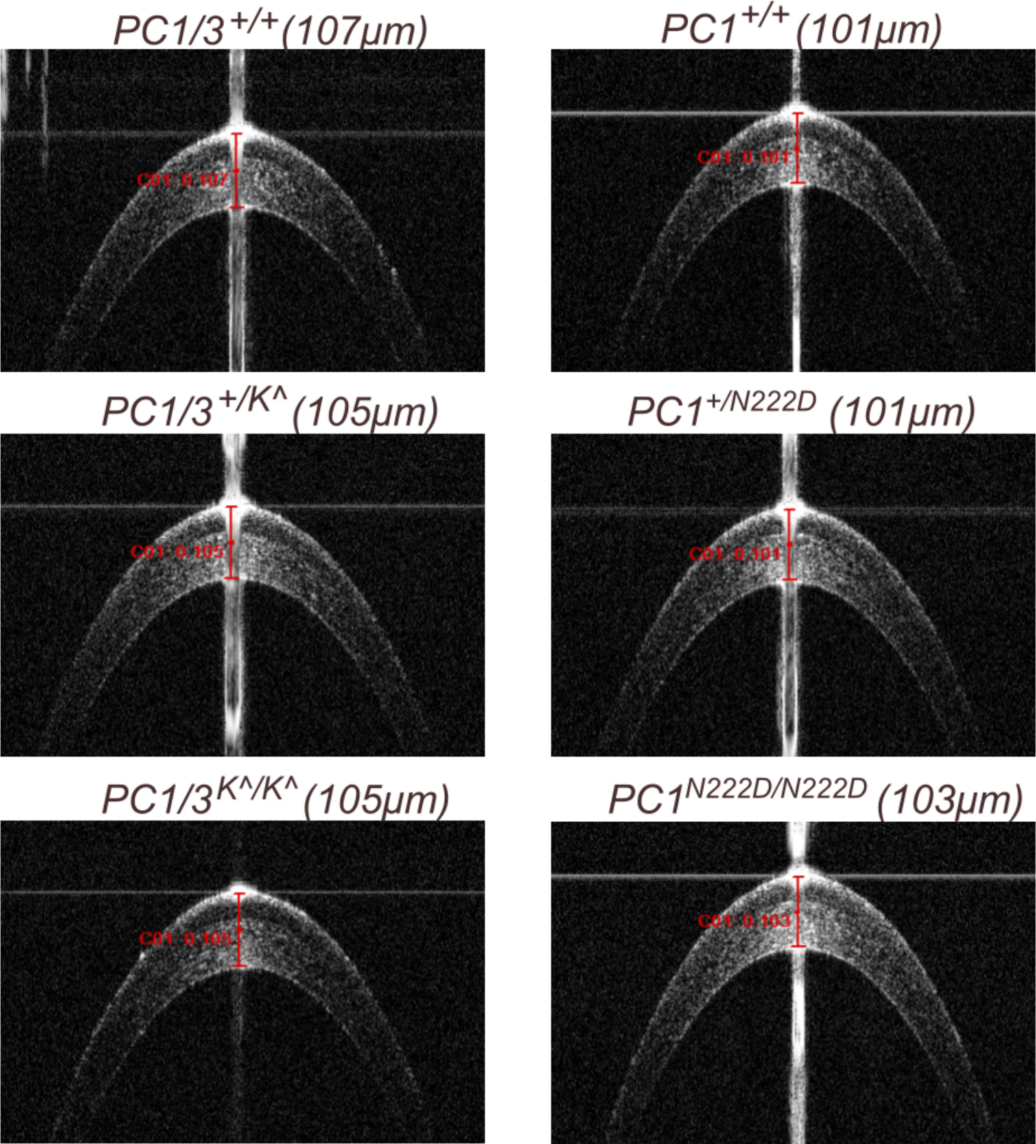
OCT images from 2 strains of *Pcsk1* mutant mice at 6 months old.

We sought to assess any differences in corneal phenotype via OCT images. Many pathological features have been reported in human patients with KC, including irregular corneal shape, anterior surface abnormalities, and peripheral corneal thinning.^36^^–^^38^ We did not identify any pathological corneal phenotypes among any genotypes of the PC1 or PC1/3 mice. Example OCT images are shown in Figure 2 for 6-month-old mice and Supplementary Figure S2 for 3-month-old mice.

### Pachymetry Evaluation in Pcsk1 Mouse Models

**Figure 3.**
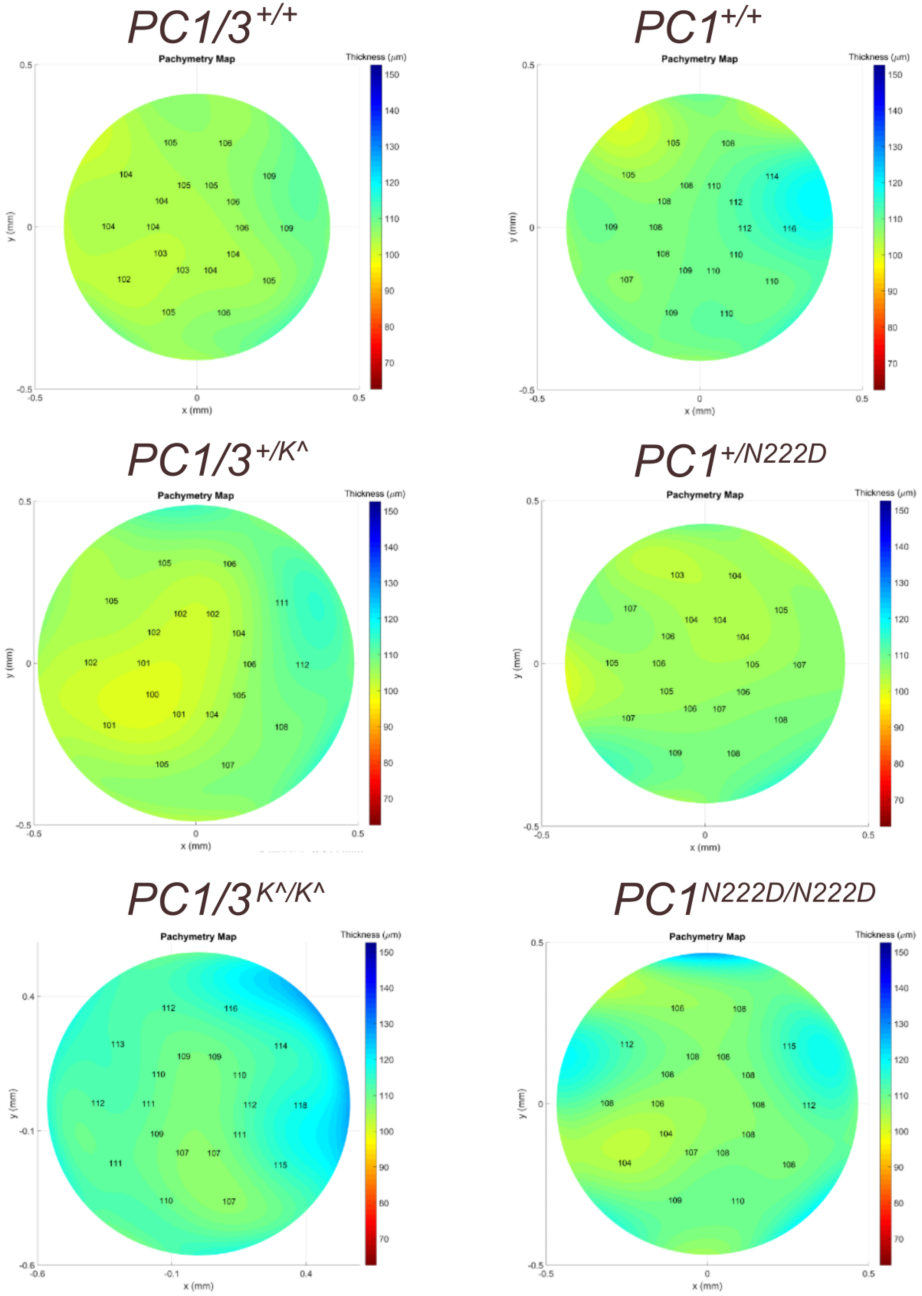
Pachymetry mapping from 2 strains of *Pcsk1* mutant mice at 6 months old.

We sought to assess differences in corneal phenotype via pachymetry corneal mapping images. Pachymetry mapping allowed assessment of the entire cornea, which is not captured by CCT measurement alone. In human patients with KC, typical features include an irregular corneal shape, anterior surface abnormalities, and peripheral corneal thinning.^37^^–^^39^ The cone in human patients with KC can be centrally or noncentrally located, necessitating evaluation of the entire cornea when determining phenotype.^40^ No pathological corneal phenotypes were identified among any of the two mouse strains of Pcsk1. Example images of corneal thickness mapping are shown in Figure 3.

### Histological Examination of Corneas of Pcsk1 Animal Models

**Figure 4.**
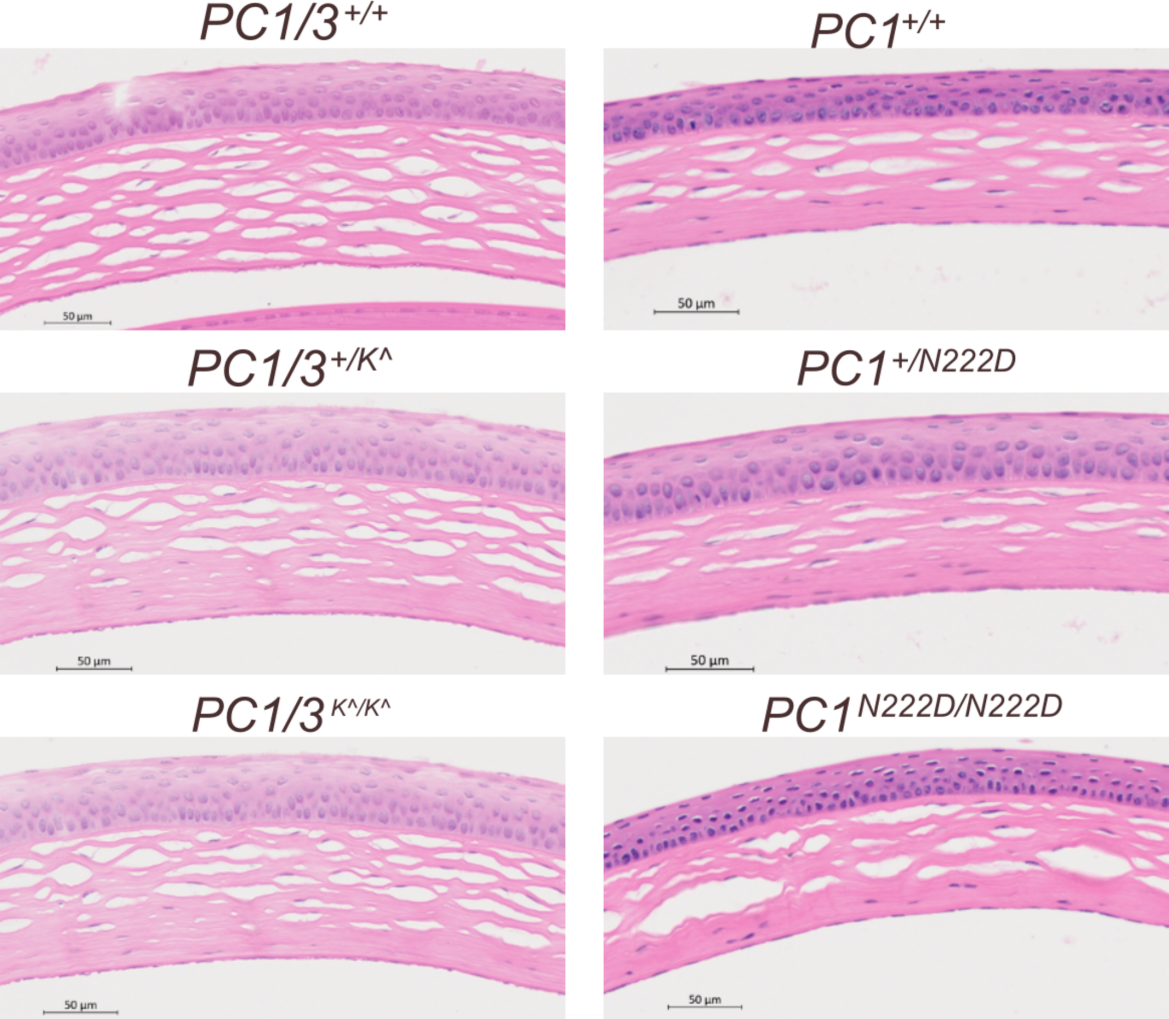
Histological staining (H&E) images of 2 strains of *Pcsk1* mutant mice at 6 months old.

We then sought to evaluate histopathological differences in mouse corneas consistent with those observed in human corneas affected by KC. Many histopathological features have been reported in human patients with KC, including abnormal epithelial thickness, interrupted basement membrane, and structural changes or fibrosis of the stromal layer.^3^^,^^41^^,^^42^ We examined four corneas from each genotype (*PC1/3^+/+^*, *PC1/3^+/K^^*, *PC1/3^K^/K^^*, *PC1^+/+^*, *PC1^+/N222D^*, and *PC1^N222D/ N222D^*) using H&E staining and did not identify any significant histological abnormalities in heterozygous or homozygous mutant mice of *Pcsk1^tm1Dfs^* and *Pcsk1^N222D^* mouse corneas (Fig. 4).

## Discussion

Mutations in the *PCSK1* gene have been associated with obesity, malabsorptive diarrhea, hypogonadotropic hypogonadism, and impaired regulation of plasma glucose levels in humans.^30^ In mice, the *Pcsk1* gene encodes PC1/3 and PC1. PC1/3, or prohormone convertase 1/3, is a neuroendocrine-specific member of the subtilisin-like proprotein convertase family.^29^ Homozygous PC1/3 KO mice are smaller in size than wildtype littermates, lack mature GHRH, and have reduced levels of pituitary growth hormone (GH). The PC1/3 KO homozygotes exhibit hyperproinsulinemia but maintain normal glucose tolerance. Heterozygote PC1/3 KO mice are mildly obese, have increased proinsulin levels, and an abnormal glucose tolerance test.^32^ PC1, also known as prohormone convertase 1 or proprotein convertase 1, is a serine endoprotease that operates on substrates such as proinsulin, proglucagon, and proopiomelanocortin (POMC).^31^^,^^43^^–^^45^ The N222D allele of mouse PC1 mimics the human PC1 mutation phenotype. This mutation leads to obesity and abnormal proinsulin processing. Mice homozygous with the N222D mutation in PC1 exhibit glucose intolerance but neither insulin resistance nor diabetes.^31^

Previous work identified the *Pcsk1* gene as a potential gene of interest connected with KC.^27^ Therefore, we sought to characterize the corneal phenotypes in 2 mouse models with varying degrees of the *Pcsk1* gene activity: PC1/3^+/+^, PC1/3^+/K^^, PC1/3^K^/K^^, PC1^+/+^, PC1^+/N222D^, and PC1^N222D/ N222D^. We observed no significant differences in CCT measurements or corneal OCT images in mutant mice compared with their wildtype counterparts at 3 and 6 months old. It has been recommended to evaluate animal models at multiple timepoints for dynamic changes.^46^^–^^48^ The corneal histological analysis is consistent with the negative OCT findings. However, the *Pcsk1* gene could contribute to the development of KC when paired with additional genetic and environmental factors.

Our study also had several limitations. First, KC is a multifactorial disease process, with environmental, hormonal, and inflammatory factors hypothesized to affect its pathogenesis.^10^^,^^12^^,^^13^ It could be beneficial to investigate the effects of genetic factors, such as *Pcsk1* gene mutations, in tandem with other contributors, such as eye rubbing or UV light exposure, to better understand the interplay among these factors in the development of KC in mice.^15^^,^^46^^,^^48^ Second, CCT may not be an outcome measure for KC in mice or human patients. Instead, elevation mapping of the posterior corneal surface could be more clinically relevant.^39^ Third, the variant in the intronic region of the *PCSK1* gene could act as a distal enhancer, regulating the expression of other genes.^27^ Fourth, it might be beneficial to examine the biomechanical properties of these mouse corneas, as patients with KC exhibit abnormal cornea biomechanics.^49^^,^^50^ Overall, we characterized two strains of Pcsk1 mice for their potential using a comprehensive characterization approach despite their nonsignificance.

## Supplementary Material

Supplement 1
