# Supplementary material for: Analysis of Keratoconus-Related Phenotypes in Two Pcsk1 Mouse Models
Source: Transl Vis Sci Technol. 2026 Feb 4;15(2):3. doi: 10.1167/tvst.15.2.3 (PMC12882103; doi:10.1167/tvst.15.2.3)
Supplement: Supplement 1 [file tvst-15-2-3_s001.docx]

**Analysis of Keratoconus-Related Phenotypes in Two Pcsk1 Mouse Models**

Carol Beatty^1^*, Jingwen Cai^2^*, Hongfang Yu^2^, Jiong Sun^1,2^, Yejin Heo^1^, Keith H. Baratz^3^, Ashlie A. Bernhisel^3^, Sanjay V. Patel^3^, Amy J. Estes^4,5^, Anthony N. Kuo^6^, Yutao Liu^2,3,4,6^

^1^Medical College of Georgia, ^2^Department of Cellular Biology and Anatomy, ^4^Department of Ophthalmology, ^5^James and Jean Culver Vision Discovery Institute, ^7^Center for Biotechnology and Genomic Medicine, Augusta University, Augusta, GA, USA. ^3^Department of Ophthalmology, Mayo Clinic, Rochester, MN, USA. ^6^Department of Ophthalmology, Duke University Medical Center, Durham, NC, USA.

*These authors contributed equally to the work

**Correspondence:**

Yutao Liu PhD, Department of Cellular Biology and Anatomy, Augusta University, Augusta, Georgia, USA; Department of Ophthalmology, Mayo Clinic, Rochester, MN, USA. E-mail: [yutliu@augusta.edu](mailto:yutliu@augusta.edu) or [liu.yutao@mayo.edu](mailto:liu.yutao@mayo.edu)

**Supplemental Figures**


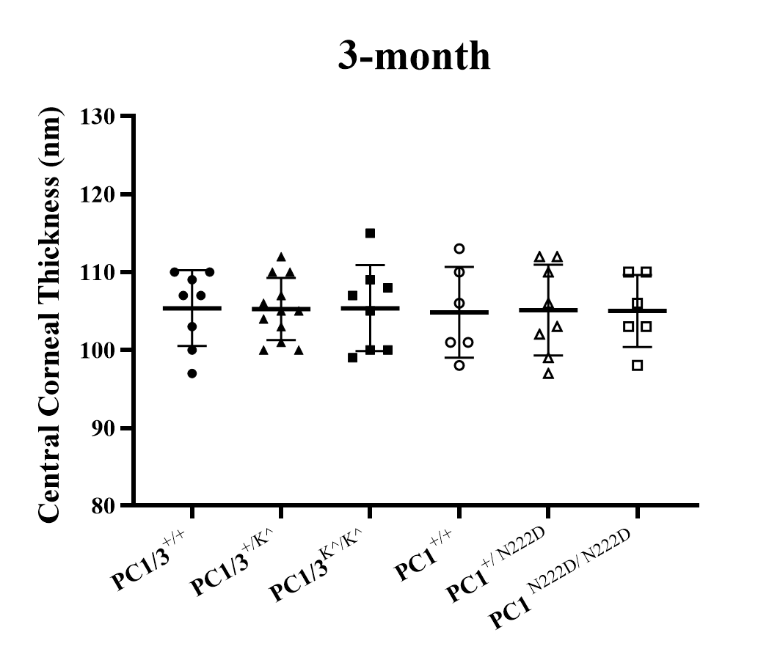


**Supplemental Figure 1**. CCT measurements in two Pcsk1 mutant strains at 3 months old. *PC1/3^+/+^* wildtype (n=4, 3 males and 1 female), *PC1/3^+/K^^* heterozygous (n=6, 6 males), and *PC1/3^K^/K^^* homozygous (n=4, 1 male and 3 females). *PC1^+/+^* wildtype (n=3, 2 males and 1 female), *PC1^+/ N222D^* heterozygous (n=4, 2 males and 2 females), and *PC1 ^N222D/ N222D^* homozygous (n=3, 3 males). There was no significant CCT difference due to genotypes in PC1/3 or PC1 strains (one-way ANOVA with Kruskal-Wallis test p-value 0.9618 and 0.9856, respectively).

| *PC1/3^+/+^* (104µm) | *PC1^+/+^* (100µm) |
| --- | --- |
| 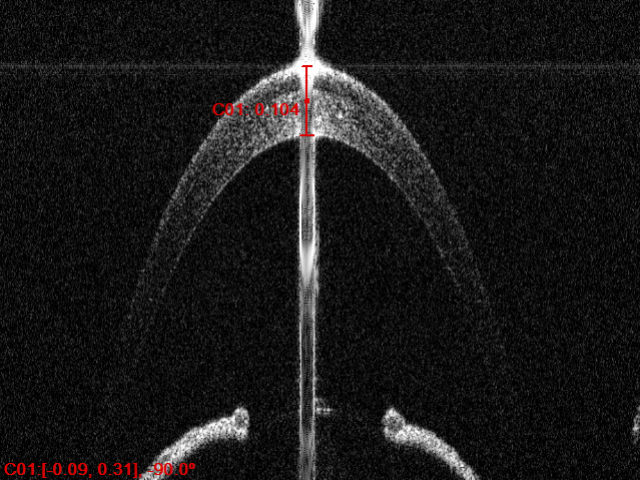 | 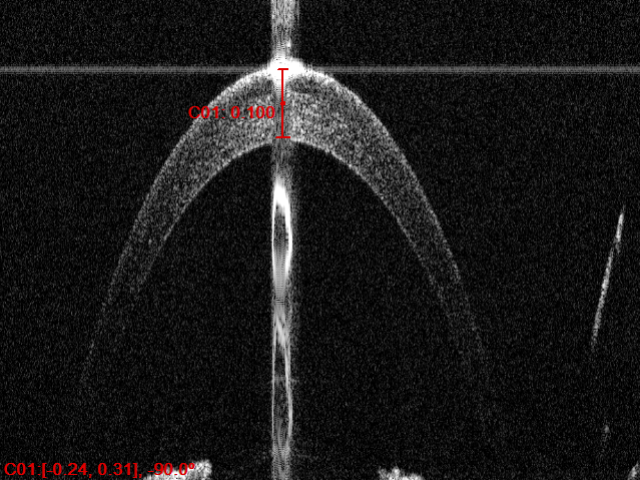 |
| *PC1/3^+/K^^* (104µm) | *PC1^+/N222D^* (104µm) |
| 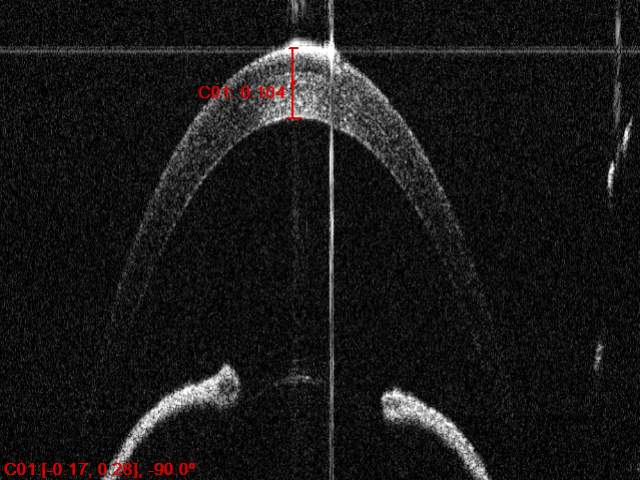 | 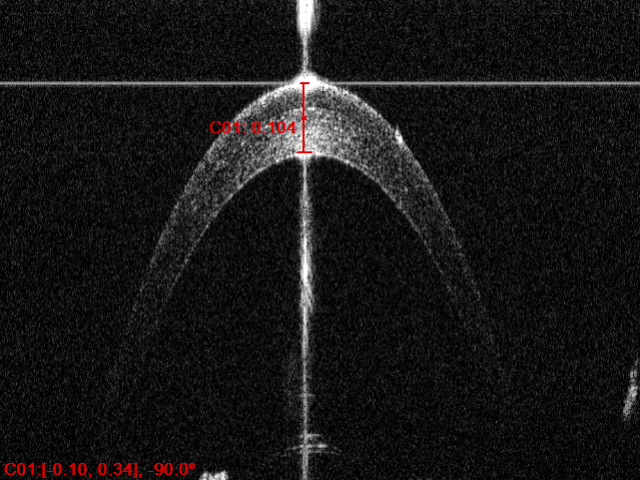 |
| *PC1/3^K^/K^^* (100µm) | *PC1^N222D/ N222D^* (106µm) |
| 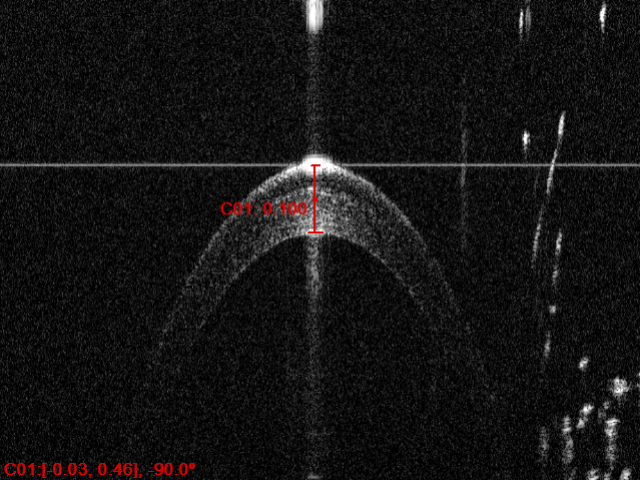 | 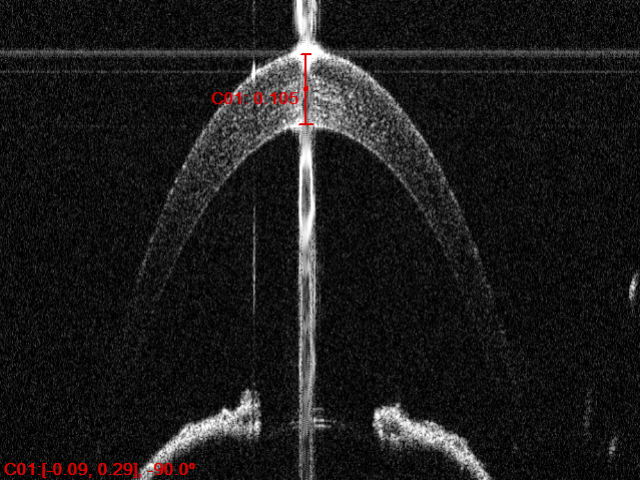 |

**Supplemental Figure 2.** OCT images from two strains of *Pcsk1* mutant mice at 3 months old.
